# Human amniotic fluid stem cells are able to form embryoid body-like aggregates which performs specific functions: morphological evidences

**DOI:** 10.1007/s00418-020-01940-3

**Published:** 2020-11-21

**Authors:** Lucia Centurione, Maria Antonietta Centurione, Ivana Antonucci, Silvia Sancilio, Gianmarco Stati, Liborio Stuppia, Roberta Di Pietro

**Affiliations:** 1 Department of Medicine and Aging Sciences, G. d’Annunzio University of Chieti–Pescara, 66013 Chieti, Italy; 2 StemTeCh Group, Center for Advanced Studies and Technology (C.A.S.T.), G. d’Annunzio University of Chieti–Pescara, 66013 Chieti, Italy; 3 Institute of Molecular Genetics, National Research Council, CNR, Unit of Chieti-Department of Medicine and Aging Sciences, University of Chieti–Pescara, 66013 Chieti, Italy; 4 Department of Psychological, Health and Territorial Sciences, School of Medicine and Health Sciences, G. d’Annunzio University Chieti–Pescara, 66013 Chieti, Italy; 5 Center for Advanced Studies and Technology (CAST), G. d’Annunzio University of Chieti–Pescara, 66013 Chieti, Italy

**Keywords:** Human second trimester amniotic fluid stem cells (hAFSCs), Embryoid body, Microvesicles, Microscopy, Cell morphology, Morphometry

## Abstract

Human second trimester Amniotic Fluid Stem Cells (hAFSCs) harbour the potential to differentiate into cells of each of the three germ layers and to form Embryoid Body (EB)-like aggregates, without inducing teratoma formation and with no ethical concerns. However, in spite of the number of reports on hAFSCs-EBs and their characterization, a thorough evaluation in light and electron microscopy of morphological and morphometric features of hAFSCs-EBs development in vitro has not been reported yet. Apart from a superficial layer of epithelial-like flat cells, displaying rare microvilli on the free surface, hAFSCs-EBs enclose inner material, abundant in vesicles and secretory granules, showing early characteristics of connective extracellular matrix dispersed among different types of inner cells. The observation of a number of microvesicles mainly represented by microparticles and, to a lower extent, by exosomes indicates the presence of a complex cellular communication system within this structure. According to morphological analysis, after 7 days of in vitro culture hAFSCs-EB appears as a well-organized corpuscle, sufficiently young to be a carrier of stemness and at the same time, when appropriately stimulated, able to differentiate. In fact, 7-day hAFSCs-EB represents itself an initial cellular transformation towards a specialized structure both in recording and in providing different stimuli from the surrounding environment, organizing structures and cells towards a differentiation fate.

## Introduction

One of the most remarkable properties of Amniotic Fluid Stem Cells (AFSCs) is their capacity to organize themselves into structures that are able to mimic some of three-dimensional (3D) properties of embryonic development. These so-called embryoid bodies represent an experimental model that has provided many important clues for unraveling early embryonic development (Brickman and Serup 2017). The formation of EBs represents the principal step in the differentiation of pluripotent embryonic stem cells (ESCs) (Valli et al. 2010). An EB is generally accepted as a characteristic self-maintaining pluripotent stem cells aggregate where cells typically “clump together”. Therefore, it is considered as a model of early embryonic development and a feature of pluripotency (Ten Berge et al. 2008; Attia et al. 2014).

This structure was originally obtained from ESCs, both human and mammalian, and thus it was called “embryoid” body. Most recently, it has been obtained from numerous types of non-embryonic stem cells or iPS, especially in humans because of ethical issues (Amos et al. 2017; Li et al. 2017; Zhu et al. 2017) and through the use of different techniques (Rungarunlert et al. 2009; Sarvi et al. 2015; Shevde and Mael 2013).

hAFSCs are considered as intermediate cells between ESCs and lineage-restricted adult progenitor cells and are able to produce EB 3D structures through the hanging drop culture method, in which cells of germ origin can be reverted to pluripotency (Desbaillets et al. 2000; Koike et al. 2007; Moschidou et al. 2013; Antonucci et al. 2014). These cell aggregates grow together as colonies and do not develop tumorigenic clusters, thereby ruling out the ethical problems related to human ESCs. EBs formation represents a principal step in the in vitro differentiation of hAFSCs that subsequently turn into three germ layers, ectoderm, mesoderm and endoderm derivatives as already demonstrated in previous studies (De Coppi et al. 2007; Antonucci et al. 2012; Di Baldassarre et al. 2018). For all these reasons, hAFSCs-EBs open up great prospects for studying the early steps of human embryonic development as well as for investigating new drugs and therapeutic strategies to correct tissue damage dysfunction. Actually, they exhibit several aspects of cell differentiation occurring during early mammalian embryonic morphogenesis, undergoing in vitro a spontaneous process recalling gastrulation (Li and Marikawa 2016). Some essential pathways for self-aggregation processes have also been identified (Valli et al. 2010). Furthermore, hAFSCs-EBs exhibit specific mesenchymal markers, such as CD90, CD105, CD73 and CD166 (De Gemmis et al. 2006; De Coppi et al. 2007), express specific genes typical of both ESCs and Primordial Germ Cells (PGCs) and are positive for specific markers of pluripotency such as OCT4 and SOX2 (Chen et al. 2012; Antonucci et al. 2014). In spite of the number of studies on in vitro cell aggregates derived from ESCs or embryonic germ cells from mouse, primates and humans, no details have been reported so far regarding the development and morphological structure and organization of hAFSCs-EBs. This could be due to the difficulty in handling and processing this relatively small 3D body of a few hundred micrometers to achieve detailed information about its structure and content.

Thus, to better investigate the hAFSCs-EBs and their possible role in cell differentiation, the aim of this study was to carry out a thorough morphological investigation with light and electron microscopy of hAFSCs-EBs after 7 days of in vitro culture. According to literature, at this time, cell aggregates are structurally organized, sufficiently “young” to be carriers of stemness and able to differentiate, when appropriately stimulated. Building on previous studies (Subra et al. 2007; van der Pol et al. 2014; Machtinger et al. 2016), we carefully analysed the internal and external surface via electron microscopy, focusing on vesicular traffic and morphological/morphometric features of different types of vesicles.

## Materials and methods

### hAFSCs collection and culture and EBs formation with hanging drop method

Briefly, to form EBs hAFSCs at the eighth passage were harvested with 0.05% trypsin and cultured in suspension in IMDM containing 15% FBS, 1 mM glutamine, 0.1 mM β-mercaptoethanol, and 1% penicillin–streptomycin (all purchased from Sigma Aldrich, Milan, Italy). 25-µl drops (1000–10,000 cells) were plated on the lid of Petri dishes (M-Medical, Milan, Italy), which were inverted onto a dish filled with 8 ml 1X phosphate-buffered saline (PBS; M-Medical) to maintain the humidity (hanging drop method). After 4 days of incubation, EBs were resuspended in the same medium and continued to grow in suspension for 2 days. After a total of 6 days in suspension, the EBs were plated onto gelatin-coated (0.1%; Sigma Aldrich) tissue culture plates for morphological analyses (Antonucci et al. 2014). Written informed consent was obtained from women in accordance with the Declaration of Helsinki. The study was approved by the ethics committee of the “G. d’Annunzio” University of Chieti–Pescara, ASL Lanciano–Chieti–Vasto, Italy and all experiments were performed in accordance with relevant guidelines and regulations.

### Alkaline phosphatase (AP) staining

Cultured hAFSC-EBs were fixed with 4% paraformaldehyde (Sigma Aldrich) for 10 min at room temperature. They were then incubated at room temperature for 2 h with 5-Bromo-4-chloro-3-indolyl phosphate/Nitro blue tetrazolium liquid substrate system (BCIP/NBT; Sigma Aldrich) to detect alkaline phosphatase activity. Samples were then washed in distilled water to remove the substrate solution and to stop the reaction.

### Immunofluorescence

hAFSC-EBs were fixed for 15 min with a 3% paraformaldehyde solution (Sigma Aldrich) at room temperature in 1X Dulbecco’s phosphate buffered saline (Sigma Aldrich), pH 7.6 supplemented with 2% saccharose (Sigma Aldrich). After cell membrane permeabilization with 0.5% Triton X-100 (Sigma Aldrich), EBs were incubated with 10% BSA (Sigma Aldrich) in 1X PBS for 30 min at room temperature, followed by a 45 min incubation at room temperature with PE-conjugated mouse anti-human OCT3/4 antibody (BD Pharmingen San Jose, CA, USA), diluted 1:500 in 1% BSA/PBS. For SOX2 (Euroclone, Milan, Italy), samples were first incubated for 60 min at room temperature with a mouse anti-human SOX2 antibody diluted 1:500 in 1% BSA/PBS. The antibodies concentration was chosen according to the manufacturer’s instructions. After rinsing three times with 1X PBS, SOX2-labelled samples were incubated for 45 min at 37 °C with a goat anti-mouse IgG-FITC (Jackson Immuno Research, West Grove PA, USA) diluted 1:100 in 1% BSA/PBS. Internal control for SOX2 was carried out by omitting only the primary antibody and did not disclose any FITC staining. Internal control for OCT 3/4 was carried out by means of isotype-matched mouse IgG PE-conjugated (Sigma-Aldrich) and did not disclose any PE staining (data not shown). After labelling, nuclei were counterstained with DAPI-mounting medium (Vector Laboratories, Inc., Burlingame, CA, USA).

### EB collection for light and transmission electron microscopy

A novel method is here reported to obtain the entire EB for a detailed study at structural and ultrastructural level, both as a whole and in section. Thus, we bypassed the embedding method used in our previous work (Antonucci et al. 2014) hard to carry out and so limited in outcoming observations. The first step of sample preparation is essential and is represented by the gentle recovery of whole small cell aggregates (EBs) from the culture plate, 7 days aged, without damaging their fragile structure. At first, each EB was individually fixed on drop for about 5 min. This passage has a double utility: on one hand, fixation undoubtedly guarantees the preservation of all the structures for better observations, on the other hand it is useful to harden the small cell aggregate, which will remain whole and intact, resisting to the light mechanical stress given by the pipetting at the time of detachment. After taking them gently, up to 10 EBs were collected in an Eppendorf and pelleted by centrifugation at 129 *g* × 8 min. From now on, pellets were never suspended and processed as described below.

### Sample preparation for light microscopy

EBs at 7 days of culture were fixed in pellet in 3% paraformaldehyde solution in 1X PBS for 15 min at room temperature, embedded in paraffin (Bio-Optica, Milano, Italy) and sectioned (2.5–3 μM) for Haematoxylin–Eosin and Mallory’s trichrome staining (Bio-Optica).

### Sample preparation for transmission electron microscopy (TEM)

Pellets were fixed with 2.5% glutaraldehyde (Electron Microscopy Science, Hatfield, PA 19,440, USA) in 0.1 M cacodylate buffer (Electron Microscopy Science), pH 7.3 for 40 min at 4 °C and post-fixed with 1% osmium tetroxide (Electron Microscopy Science) for 40 min at 4 °C. After being dehydrated in progressively higher concentrations of alcohol, samples were embedded in Spurr resin (Electron Microscopy Science) according to the method previously used in our laboratories (Centurione et al. 2010). Semithin sections (0.7–1 μM) were stained with 1% toluidine blue (Electron Microscopy Science) and analysed with a ZEISS Axioskop light microscope (Carl Zeiss, Gottingen, Germany) equipped with a Coolsnap digital camera (Photometrics, Tucson, AZ, USA). Ultrathin sections (70 nm) were cut with a Reichert ultramicrotome (Reichert, Inc., Teramo, Italy), mounted on 200 mesh copper grids (Electron Microscopy Science), counterstained with UranyLess and Lead Citrate (Electron Microscopy Science) and photographed by means of a ZEISS-109 electron microscope equipped with a Gatan 830Z00W44 camera and Digital Micrograph application used for acquiring, visualizing, analysing, and processing digital image data (Gatan GmbH, Ingolstadterstr. 12, D-80807 München, Germany).

### Light microscopy observation and morphometric analysis

The observation of samples processed for Immunofluorescence, Alkaline Phosphate, Haematoxylin–Eosin and Mallory’s trichrome staining solution was carried out with a Zeiss Axioskop 40 light microscope (Carl Zeiss) equipped with a Coolsnap Videocamera (Photometrics). MetaMorph 6.1 Software System (Universal Imaging Corp, Molecular Device Corp, CA, USA) was employed to acquire digital images and perform morphometric analysis, as already described (Antonucci et al. 2014). Morphometric computerized analysis of about 100 EBs mean diameters and the specific vesicle areas and diameters (10 photographic fields at 20,000 × on 5 sections) were performed manually drawing the area regions and the diameter segments, after calibrating the MetaMorph 6.1r6 program (Universal Imaging Corp) for the magnification used (5 ×; 20000 ×). MetaMorph numeric data were logged to Microsoft Excel to calculate mean values and standard deviation (SD).

## Results

**Fig. 1:**
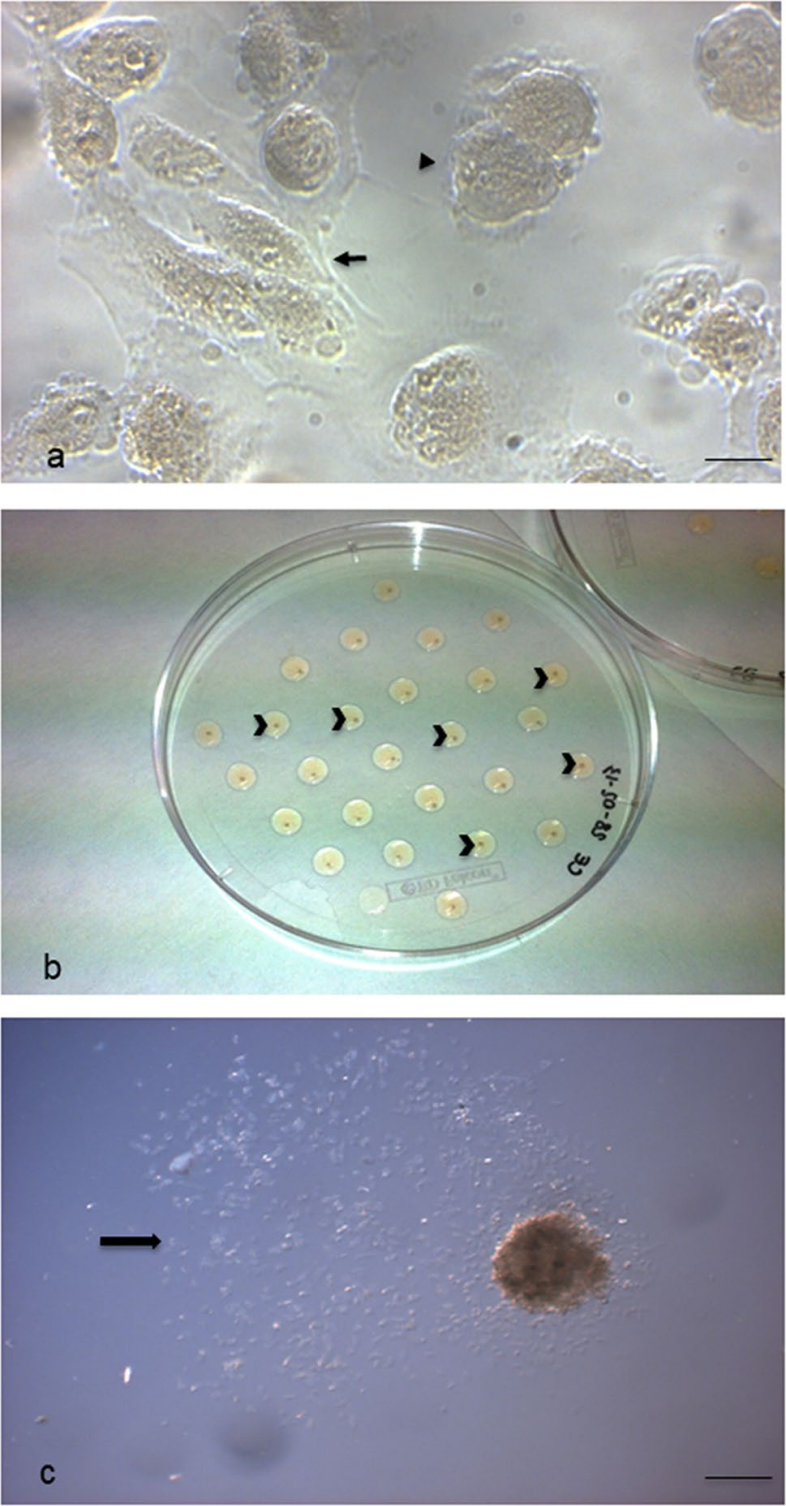
Embryoid Bodies (EBs) formation. **a** Cultured hAFSCs observed under phase contrast microscopy. Cells with different morphological features are evident: fibroblast-like (arrow) and epithelial-like (arrowhead) cells (scale bar: 14 μm). Magnification: 20 ×. **b** 7-day-cultured EB through the hanging drop method in overturned uncoated Petri dish for sampling. Each small and light brown EB corpuscle is contained in a drop (arrowheads). **c** 7-day-cultured EB observed under phase contrast microscopy. The 3D agglomerate appears flattened and with an irregular surface; many cells are evident around it (arrow) (scale bar: 206 μm). Magnification: 5 ×

Isolated pluripotent hAFSCs cultured in vitro showed typical morphological patterns of both fibroblast-like and epithelial-like cells (Fig. 1a), as we already described (Centurione et al. 2010; Pipino et al. 2015). hAFSC-derived EBs cultured in vitro for 7 days appeared as spherical and compact agglomerates, macroscopically evident, with the mean diameter of 372.4 μm (Fig. 1b). These microscopic and morphometric evaluations were performed using an inverted phase contrast microscope, through which the 3D organization of the corpuscle was confirmed, although it appeared rather flattened and without sharp edges on the surface (Fig. 1c). Interestingly, the EB size was achieved already after 3 days of culture (data not shown).

**Fig. 2:**
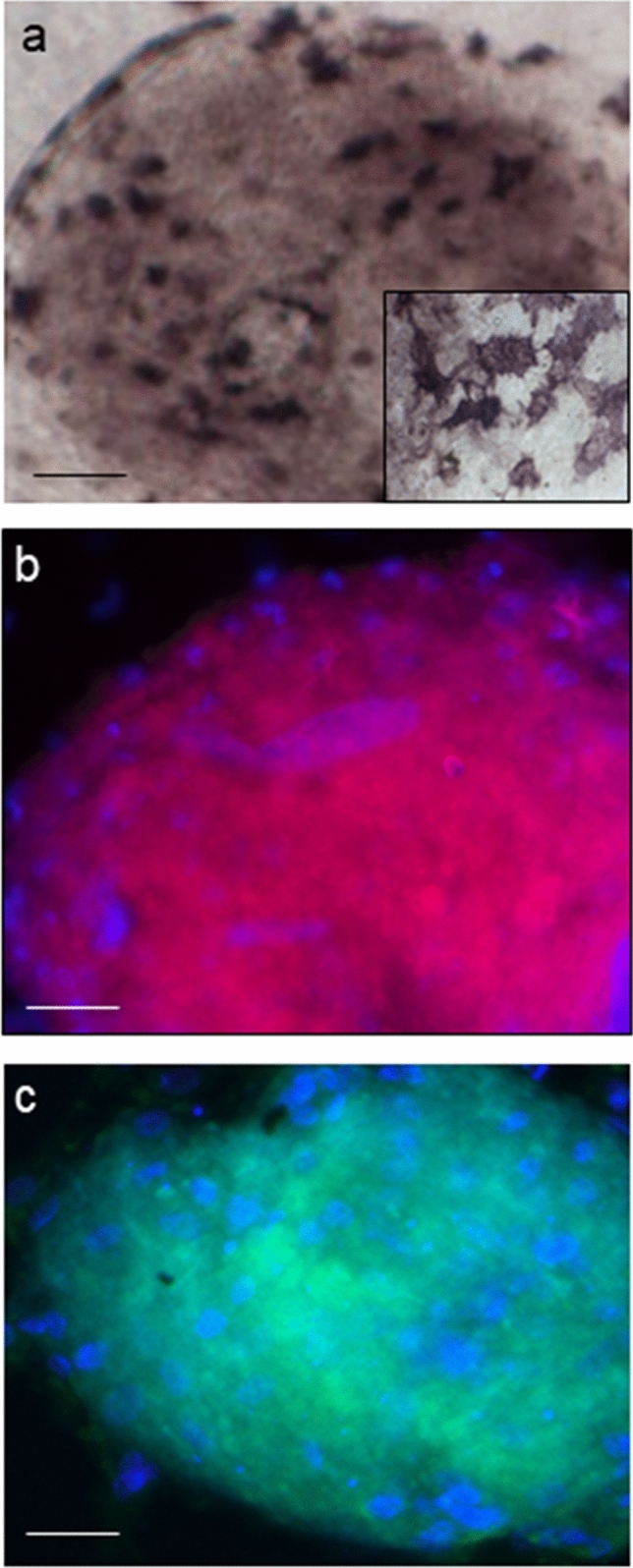
Entire EB specific markers of stemness and pluripotency. **a** Alkaline Phosphatase histochemical detection (scale bar 32 μm). Magnification: 20 ×, inset: 40 ×. **b** SOX2 red immunofluorescence (scale bar: 32 μm). Magnification: 20 ×. **c** OCT3/4 green immunofluorescence (scale bar: 32 μm). Magnification 20 ×. **b, c** Nuclei were counterstained with 6-diamino-2-phenylindole (DAPI) (blue fluorescence)

As expected, hAFSC-derived EBs were positive to well-known stemness markers, such as Alkaline Phosphatase (Fig. 2a), OCT3/4 (Fig. 2b) and SOX2 (Fig. 2c).

**Fig. 3:**
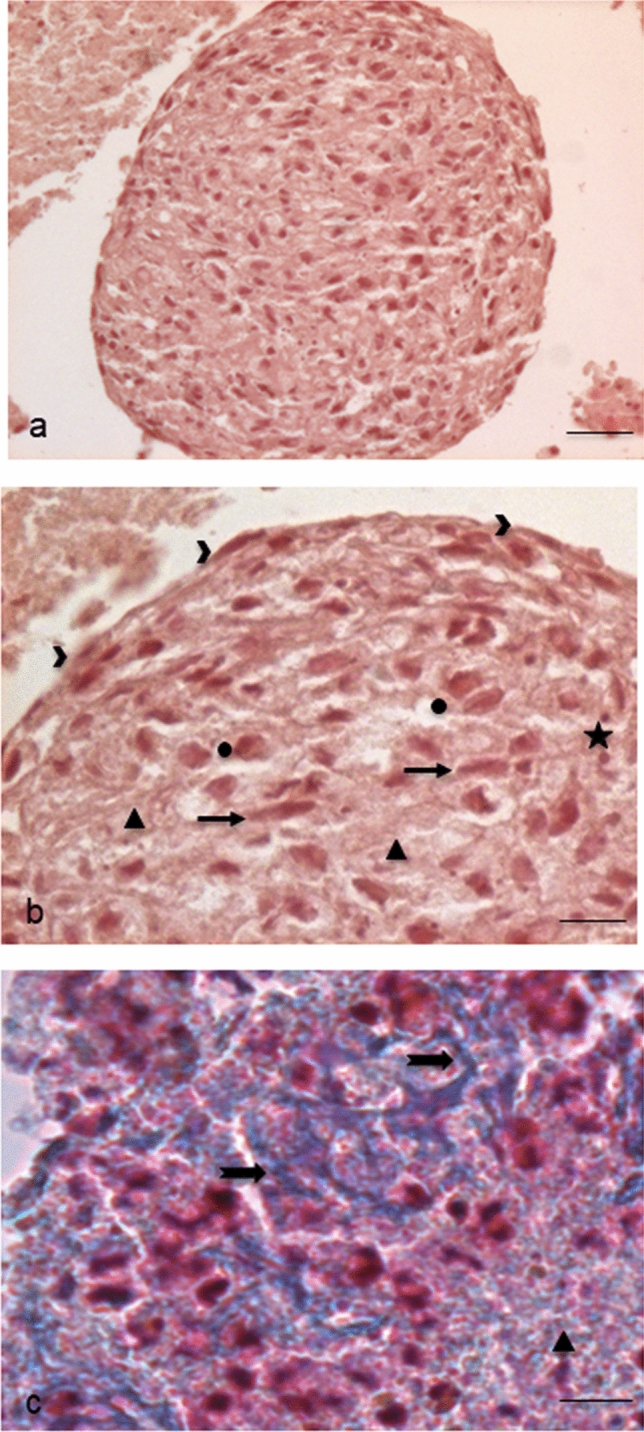
Sections of paraffin-embedded EBs for Light Microscopy. **a** Haematoxylin–Eosin stained EB whole section (scale bar: 44 μm). Magnification: 10 ×. **b** Enlarged detail of the same EB: we can observe on the surface flattened cells (arrowheads) and inside different types of cells such as elongated fibroblast-like cells (arrows), more roundish and widened cells (circles), apoptotic cells and micronuclei (stars). Extracellular matrix (triangles), at times rarefied, shows the presence of fibrillar material (scale bar: 11 μm). Magnification: 40 ×. **c** Mallory’s Trichrome nicely reveals the blue-stained connective extracellular matrix (triangles) and the early organization of connective fibres (arrows) (scale bar: 11 μm). Magnification: 40 ×

To investigate in detail the structure and the possible content of this cellular aggregate, we first proceeded with sectioning and evaluating paraffin-embedded EBs. Haematoxylin–Eosin stained sections showed a roundish cellular aggregate (Fig. 3a), surrounded by flattened epithelial-like cells on the surface and containing inside an amorphous rarefied material mixed with elongated fibroblast-like cells, round cells, apoptotic cells and cellular debris (Fig. 3b). The amorphous material showed a slightly blue colour with the Mallory’s trichrome staining, thus revealing the presence of connective-like matrix in early deposition (Fig. 3c).

**Fig. 4:**
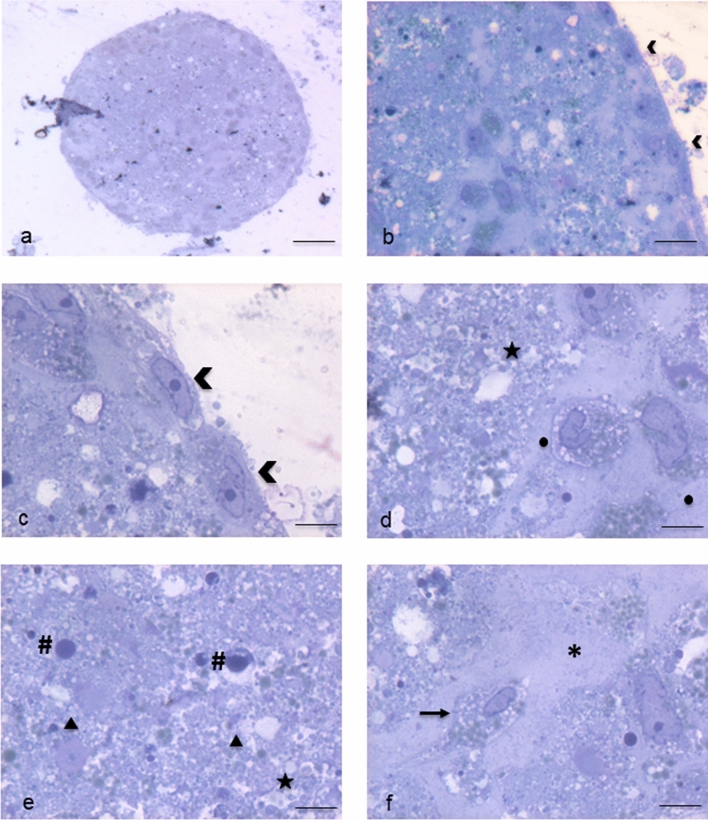
Spurr resin-embedded EB, semithin sections for Light Microscopy. **a** EB fully-rounded structure (scale bar: 46 μm). Magnification: 10 ×. **b** On the outer surface EB is covered with a monolayer of flattened epithelial-like cells (arrowheads) (scale bar: 12 μm). Magnification: 40 ×**c** Euchromatinic nuclei and prominent nucleoli in superficial cells (arrowheads) (scale bar: 4.6 μm). Magnification: 100 ×. **d** Deeper rounded cells (circles) surrounded by fibrillar substance, containing secretory granules (star) (scale bar: 4.6 μm). Magnification: 100 ×. **e** Apoptotic cells and micronuclei (hash marks) dispersed in an amorphous granular (stars) material (triangles) (scale bar: 4.6 μm). Magnification: 100 ×. **f** Fibroblast-like cell (arrow) surrounded by fibrillar material (asterisk) (scale bar: 4.6 μm). Magnification: 100 ×

**Fig. 5:**
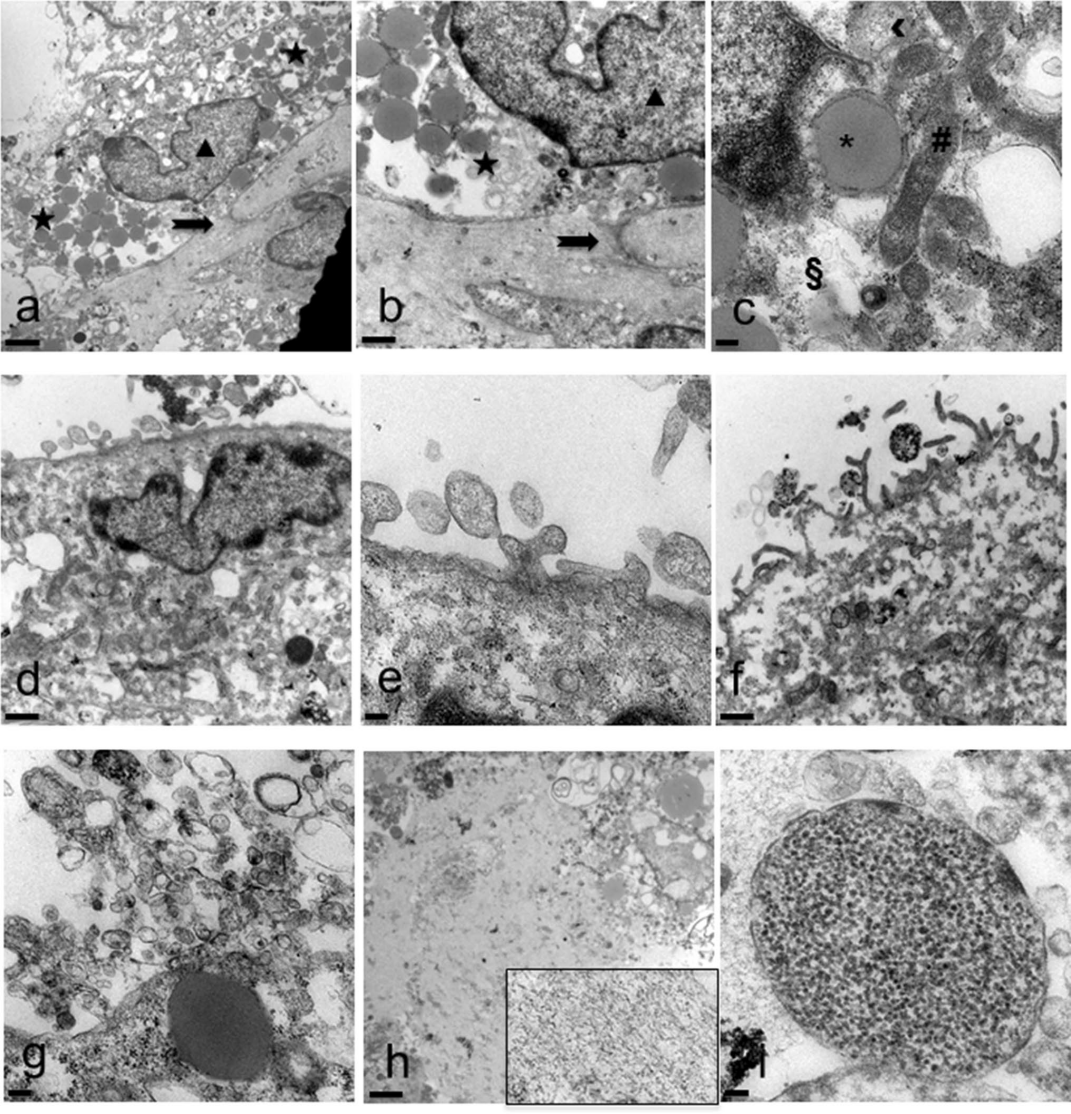
Spurr resin-embedded EB, ultrathin sections for Transmission Electron Microscopy. **a** Superficial cell with euchromatic nucleus (triangle) and cytoplasm rich in granules (stars) of different aspect. Cell junctions in primitive organization are observed (arrow) (scale bar: 2 μm). Magnification: 3000 ×. **b** Detail at higher magnification (scale bar: 1 μm). Magnification: 7000 ×. **c** Cytoplasm rich in organelles: mitochondria (hash mark), ribosomes (double S), rough and smooth endoplasmic reticulum (arrowhead). Large lipid granule surrounded by membrane (asterisk) (scale bar: 0.2 μm) Magnification: 20,000 ×. **d** Cell surface membrane shows gemmation of different types of vesicles (scale bar: 1 μm). Magnification: 7000 ×. **e** Detail at higher magnification (scale bar: 0.2 μm). Magnification: 20,000 ×. **f** Evident surface microvilli (scale bar: 1 μm). Magnification: 7000 ×. **g** Numerous vesicles of different size and shape (scale bar: 0.2 μm). Magnification: 20,000 ×. **h** Inner fibrillar matrix content (better visible in the inset magnified 20,000 ×) (scale bar: 2 μm). Magnification: 3000 ×. **i** Dispersed in the inner material is appreciable a vesicle containing a granular substance (scale bar: 100 μm). Magnification: 50,000 ×

**Fig. 6:**
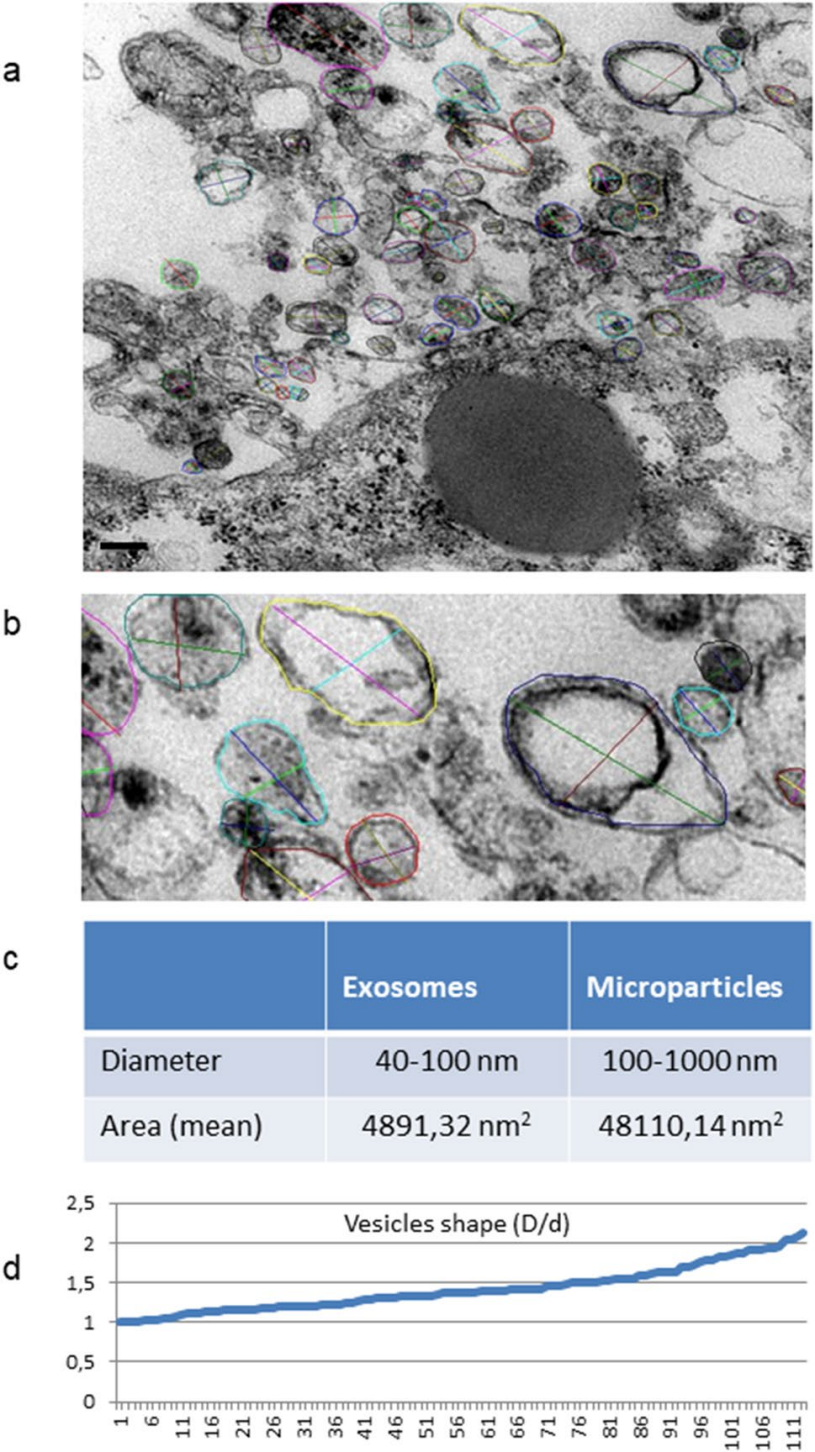
Ultrastructural, morphological and morphometric evaluation of superficial extracellular vesicles. **a** Numerous vesicles with different size and content are visible on the EB surface (scale bar: 0.2 μm). Magnification: 20,000 ×. **b** Detail at higher magnification. The coloured circles mark the measured areas and the coloured segments represent the measured largest and minor diameters of each vesicle. Magnification: 40,000 ×. **c** The table shows the percentage of exosomes and microparticles ranked according to their size. **d** Graph of the numeric evaluation of vesicles shape, from roundish to oval; here is shown a sample of about one hundred microvesicles analysed (*X*-axis); the ratio between the largest diameter *D* and the minor diameter *d* (*Y* axis) ranges from 1 to 2.13

Electron microscopy highlighted in detail the peculiarities of cell membrane exposed surface and cytoplasmic content as well as of the amorphous substance located inside the EB. At first, semithin sections showed the full round shape of EB and confirmed the surface arrangement of flattened cells, mainly in a single layer, displaying euchromatic nuclei and prominent nucleoli (Fig. 4a, b). The inner amorphous content was rich in small gaps, merging sometimes into a larger single cavity, and secretory granules of different appearance, some of which observed both outside the cells and in the cytoplasm (Fig. 4c, d). Dispersed in the inner material were also present round shaped cells, rich in cytoplasmic secretory granules, surrounded by a thinly fibrillar connectival matrix and some apoptotic cells with their electron dense micronuclei (Fig. 4e). Some inner cells displayed the presence of cytoplasmic extensions (Fig. 4f). Ultrastructural analysis of EB superficial cells revealed an abundant cytoplasm rich in organelles such as mitochondria, ribosomes, endoplasmic reticulum and granules/vesicles of different size and content, all unmistakable signs of intense metabolic activity; they showed also cell junctions in primitive organization (Fig. 5a–c). On the outer cell membrane rare microvilli surrounded by different types (in content electron density, shape and size) of extracellular vesicles (EVs) were visible (Fig. 5 d–g). Inside the EB, dispersed matrix confirmed its fibrillar structure (Fig. 5h). Furthermore, we analysed the size of superficial EB extracellular vesicles **(**Fig. 6a, b), to verify the presence of microparticles and/or exosomes by differentiating them according to range dimensions (Fig. 6c). Interestingly, according to morphometric measures we found that the vast majority of the vesicles analyzed (73%) were microparticles, whereas the remaining 27% of the vesicles could be classified as exosomes. The numeric evaluation of all vesicles shape from roundish to oval, that is the ratio between the largest diameter and the minor diameter, revealed a predominance of round shapes compared to oval ones (about 70%).

## Discussion

Human Embryoid Body is a structure that initially has been studied and in vitro obtained from ESCs, whose certain pluripotent potential was also demonstrated by EBs developing towards the expected three germ layers (Lai et al. 2015). hAFSCs possess intermediate characteristics between embryonic and adult stem cells, are able to form EBs and show a pluripotent potential. Furthermore, unlike ESCs, they do not imply ethical issues, are non-teratogenic and non-immunoreactive (Moschidou et al. 2013; Gholizadeh-Ghaleh Aziz et al. 2019). Here we report for the first time a thorough morphological in situ analysis of human EBs at 7 days, a useful age to observe a structure already partially organized, but sufficiently young to be still able to differentiate. Our detailed morphological and morphometric study revealed that 7-day hAFSC-EBs indeed represent themselves an initial cellular transformation step towards differentiation. Recently, morphological studies of the structures, and, therefore, the understanding of the function, have recently aroused great interest in researchers. In fact, they can closely correlate cell shape and, therefore, cytoskeletal organization, with the resulting biochemical stimuli leading to the fate of the stem cell during differentiation (von Erlach et al. 2018; Pourquié 2018). Moreover, it was stated that the different cellular shapes are found to be carriers of spatial information to guide fundamental processes for multicellular organization and cell functions during development (Haupt and Minc 2018). EB, in fact, was found to be an in vitro 3D cell aggregate that, once formed, explains the greater differentiation capacity of cells and their features related to pluripotency, also documented by a group of transcription factors and markers of stemness and pluripotency like Alkaline Phosphatase, SOX2 and OCT4 (Moschidou et al. 2012). Its spherical shape also displays a minimum surface/volume ratio, i.e., it contains a reduced exposed surface and a greater volume, which increases more than the surface (Haupt and Minc 2018) to contain and protect inner material, but, at the same time, to interact with stimuli coming from outside and ready to be outsourced. The superficial epithelial-like EB cells, in fact, remain exposed to external surface with a free border with microvilli to absorb substances or to exchange secretory products and stimuli. On the other side they tend to stay in contact with each other through primitive junctions and with deeper cells and/or with the inner amorphous content rich in fibrillar extracellular matrix and secretions continuously produced (also by roundish and granular inner cells) and exchanged inside. The secretions produced remain at the disposal of the cells themselves, which are repeatedly stimulated even from the inside, to grow and/or differentiate, with an autocrine or paracrine mechanism. Furthermore, the observed presence in all the cells of many cytoplasmic organelles is a sign of the strong metabolic activity of the cells themselves. Accordingly, the presence of granules of various nature and size found in the cytoplasm and dispersed in the amorphous content gives reason for the intense secretory activity attributable to cells that metabolically respond to stimuli and produce effects on neighbouring cells. Today, in fact, for in vitro studies, researchers are interested in studying the effects of the stem cells culture "conditioned" medium, where all the secretions are collected and where their great potential is concentrated (Alves da Silva et al. 2015; Haneef et al. 2014; Balbi et al. 2017; Vizoso et al. 2017).

Microparticles and exosomes are the most widely studied and best-known extracellular vesicles types present in body fluids. They are released in the microenvironment to play a wide range of regulatory functions such as coagulation, inflammation, cellular homeostasis and survival, intercellular communication, transport and some types of differentiation. Many efforts are necessary to study their nature, content and functions, including size and morphology (Nieuwland and Sturk 2010; van der Pol et al. 2010–2014; Raposo and Willem 2013; Vestad et al. 2017; Angelini et al. 2016). Via TEM observation and morphometric analysis, following the range of dimensions reported in literature, we were able to identify and quantify the presence of both types of extracellular vesicles, expressed as a percentage. Moreover, the numerical calculation of the ratio between the larger and smaller diameter of the EVs enabled us to identify the precise shape of these very small structures, smaller than one micrometer. Based on our observations, we can assume that, as time passes, a sort of spontaneous evolution and development of hAFSCs-EBs occurs. In fact, we observed that EB is firstly a mere cellular aggregate (at 3 days of culture, data not shown) that at 7 days of culture forms an organized corpuscle coated with a monolayer of flattened and adherent cells delimiting an organic and secretory content, in which different types of roundish and fibroblast-like cells are dispersed, some of which surrounded by a thinly fibrillar substance, to become at 14 days of culture a multi-layered wall structure with a large hollow inside, where secretions greatly increase in amount, being localized even in cellular spaces as we previously demonstrated (Antonucci et al. 2014).

In conclusion and in agreement with previous studies, we suggest that in vitro hAFSCs-EB represents the best structure for cell-to-cell and outside and inside world interaction to obtain cell differentiation for the formation of human three germ layers and for regenerative medicine purposes. In fact, cells remain exposed to their own secretions and interactions, accepting and releasing messages in a microenvironment, protected by an external thin coating, specialised for exchanging. In the inner portion, the corpuscle is always evolving, ensuring sufficient space to collect and preserve secretions as well as differentiating cells, allowing the pursuance of events and heading their carrying out. Interestingly, a similar corpuscle was recently obtained with a stem-cell-based protocol from human ESCs and named “the organizer” of the developmental processes (Pourquié 2018). However, hAFSCs-EBs could represent a useful laboratory model both to induce stimuli for differentiation in different types of stem cells and to study with no ethical implications the numerous related processes that could resemble early stages of human development.
